# A systematic mixed-integer differential evolution approach for water network operational optimization

**DOI:** 10.1098/rspa.2017.0879

**Published:** 2018-09-05

**Authors:** Wanqing Zhao, Thomas H. Beach, Yacine Rezgui

**Affiliations:** Cardiff School of Engineering, Cardiff University, Cardiff CF24 3AA, UK

**Keywords:** potable water distribution, differential evolution, network operation and control, hydraulic model, optimization

## Abstract

The operational management of potable water distribution networks presents a great challenge to water utilities, as reflected by the complex interplay of a wide range of multidimensional and nonlinear factors across the water value chain including the network physical structure and characteristics, operational requirements, water consumption profiles and the structure of energy tariffs. Nevertheless, both continuous and discrete actuation variables can be involved in governing the water network, which makes optimizing such networks a mixed-integer and highly constrained decision-making problem. As such, there is a need to situate the problem holistically, factoring in multidimensional considerations, with a goal of minimizing water operational costs. This paper, therefore, proposes a systematic optimization methodology for (near) real-time operation of water networks, where the operational strategy can be dynamically updated using a model-based predictive control scheme with little human intervention. The hydraulic model of the network of interest is thereby integrated and successively simulated with different trial strategies as part of the optimization process. A novel adapted mixed-integer differential evolution (DE) algorithm is particularly designed to deal with the discrete-continuous actuation variables involved in the network. Simulation results on a pilot water network confirm the effectiveness of the proposed methodology and the superiority of the proposed mixed-integer DE in comparison with genetic algorithms. It also suggests that 23.69% cost savings can be achieved compared with the water utility's current operational strategy, if adaptive pricing is adopted for all the pumping stations.

## 1. Introduction

In the operation of potable water distribution networks [1], energy cost from pumping accounts for the major proportion of total expenditure [2–4]. The network operation, including pump and valve control, is often managed by experienced field engineers using heuristics in the form of local operating rules (e.g. the on/off control of a fixed-speed pump in response to a reservoir's real-time water levels). This can be realized by the use of supervisory control and data acquisition system or distributed control system. A feedback control loop is normally formed between sensors, controllers and actuators all located in the field, while a water utility's control centre (e.g. a centralized regional telemetry system) may exist to remotely monitor the functioning of the overall system with data transmitted via field controllers. Different actuators involved in the water network are usually managed separately, without fully exploiting the interrelationships between one another in conjunction with other factors such as the complexity of energy tariff and the physical structure and characteristics of the network. Achieving a water network's primary goal of supplying a sufficiently high tap pressure without a systematic view of the network operation, can thus result in unnecessarily wasted energy expenses on pumping.

Therefore, to save energy costs, the network operator needs to consistently seek opportunities to (i) achieve a minimum pressure head loss across the network, (ii) operate pumps at a high degree of efficiency (dynamically determined by the intersection point of the pump curve and the time-varying system curve), (iii) factor in the complexity of the energy tariff, and (iv) meet various types of operational and hydraulic constraints. Given these complex, dynamic and multidimensional considerations, a challenging operational optimization problem is presented for the real-time management of water distribution networks. The essential research question then lies in how to produce a holistic cost-effective operational strategy that can deliver a systematic and real-time control of the water network, while accommodating network characteristics and statuses, consumer demand profiles, energy tariffs and a series of hydraulic and operational constraints.

As has been successfully applied in a wide range of domains, traditional optimization methods including linear programming [2] (based on the linearization of hydraulic constraints), dynamic programming [5] and nonlinear programming [3] (based directly on nonlinear models), were among the early attempts to perform water network operational optimization. They are designed to reduce energy and/or pump maintenance costs by optimized scheduling of pumps, operating for every time interval (e.g. 1 h) within a given operational horizon (e.g. 24 h), also commonly being referred to as pump scheduling. Particularly, the minimization of energy cost over an operational horizon considering constraints of water hydraulics, nodal pressures and tank levels was usually pursued. Although these traditional methods are able to quickly find operational solutions, they generally suffer from poor optimization performance and/or oversimplified networks. Moreover, these works are often site-specific and as such not scalable, i.e. difficult to transfer the derived technologies for operation on a different water network.

With the great prevalence of metaheuristic methods applied in diverse domains and scenarios, some have also been adopted for the operational optimization of water networks without simplifying the underpinning hydraulic models. These include genetic algorithms (GAs) [6–8], simulated annealing (SA) [9,10], particle swarm optimization (PSO) [11] and ant colony optimization (ACO) [12,13]. To define pump schedules, binary encoding is most commonly adopted, where only fixed-speed pumps are involved in the network and their on/off states across different time intervals can thus be represented by several binary bits. Alternatively, time-controlled triggers [14,15] can also be used, where the active and inactive periods of time intervals resulting from every pump switch are successively coded in an individual solution. On the other hand, real-value encoding is used to directly define the actuator's continuous states for water networks where only variable-speed pumps are involved, though this has attracted significantly less research. Furthermore, multi-objective optimization methods have also been used for the optimization of energy cost, maintenance cost and/or reservoir level variation simultaneously, including non-dominated sorting genetic algorithm (NSGA), NSGA-II, strength Pareto evolutionary algorithm (SPEA), SPEA2 and niched-Pareto genetic algorithm (NPGA) [16–18]. Although promising research results have been achieved, their real-time applications are somewhat limited due to the high computational burden and the complexity of quickly presenting and selecting solutions for network operators.

A well-defined methodology for the network operational optimization was proposed in the potable water distribution management POWADIMA research project [19–21], assessed by a modified Anytown benchmark network and two pilot networks (i.e. the Haifa-A network in northern Israel and Valencia network on the eastern seaboard of Spain). In the proposed methodology, artificial neural networks were developed to capture the domain knowledge of water networks and were then embedded in the optimization process to efficiently predict the outputs of each combinatorial strategy for all the time intervals forming the operational horizon. The operational strategy was coded as an individual solution within GAs and was evolved for better performance through a number of generations. A multiplicative penalty method was used to deal with the constraints when calculating the fitness value for all GA solutions.

Recently, the branch-and-bound algorithm [22] based on a divide-and-conquer strategy was applied to pump scheduling. Puneet & Larry [23] also introduced an optimization model based on a commercial solver (AMPL) for optimal pump operation considering uncertain demands. In conclusion, as in previous studies, either fixed-speed or variable-speed pumps are considered for network operation. The reality is that both fixed-speed and variable-speed pumps can simultaneously exist in a water distribution network. Moreover, the optimization is often static and is conducted daily (sometimes more frequently) to produce the network operational strategy usually for the following day (which is thereby referred to as an operational or planning horizon of 24 h). This static strategy generating mechanism can cause problems including accumulated prediction errors and violated constraints. This is because the discontinuous connection between the day-to-day alike optimization can lead to some constraints (especially reservoir levels) dramatically violated in the following day no matter how the operational strategy is adjusted, due to bad network statuses resulting from the previous day of optimization. In other words, a previous day's optimization does not foresee the potential output from the following day. Extra constraints would then need to be introduced, for example, the reservoir level at the end of the day should be higher than that at the beginning, inevitably increasing the optimization burden and decreasing the network performance.

Differential evolution (DE) as a promising metaheuristic for continuous optimization has recently seen its applications in the optimal design of water distribution networks [24–28]. The goal is to minimize the network design cost, usually through the determination of pipe, pump and reservoir sizes. For instance, Zheng *et al.* [27,28] proposed multi-stage approaches involving the decomposition of the water network using graph algorithms and then the optimization of the decomposed networks using linear and nonlinear programming and DE. However, the applications of DE in finding optimal control strategies for the network operational problem are still rarely seen in the water research community. In this paper, a systematic methodology based on DE and model-based predictive control is proposed for the operational optimization of potable water distribution networks. Our methodology forms a feedback control loop between field sensors, a central controller and field actuators. The operational strategy is periodically computed in the central controller considering a finite rolling horizon (i.e. optimization horizon) of predicted field statuses and is then sent to the field actuators, as long as a set of monitoring data is received from the field sensors. By moving from the planning to optimization horizon, a finite horizon of future network statuses can thus always be taken into account by the optimization process. The dynamic change of the water network is thereby factored given the successive operational optimization and update of control strategies. A mixed-integer DE algorithm is then devised to perform the actual optimization tasks defined within the underlying control problem, while being able to deal with both continuous and discrete actuations variables, as well as actuator, hydraulic and operational constraints. The whole optimization methodology is developed as a C library file for its flexibility of integration across different water networks. A water utility developed hydraulic model from a real site is finally used to demonstrate the effectiveness of the proposed methodology in different settings.

Therefore, the contribution and novelties of the present work can be summarized into two perspectives. (i) A systematic optimization methodology is designed for the real-time operation of water networks by dynamically producing control strategies, while being able to holistically take into account various complexities and constraints (e.g. network characteristics, energy tariff, consumption profiles and nodal demand pressure). Such a control strategy is periodically optimized for actuation considering a finite rolling horizon of predicted network statuses upon the updating of the field monitoring. (ii) A novel adapted mixed-integer DE algorithm is devised with the ability to handle both continuous and discrete decision variables simultaneously (e.g. fixed and variable speed pumps) in a water network. The performance is also validated in a pilot water network in the UK and demonstrated to outperform that of GAs in terms of increased cost savings and less computational time.

The paper is organized as follows. Section 2 presents the preliminaries of water network operation including the objective and constraint formulation. The mechanism of DE is then briefly described in §3. Section 4 proposes the methodology for water network operational optimization including the model-based predictive control scheme, the encoding strategy and the mixed-integer DE algorithm. A pilot area is then described in §5, followed by the simulation results and analysis from applying the proposed methodology. Finally, §6 concludes the paper.

## 2. Preliminaries

The major energy cost within a potable water distribution network arises from pumping stations, which account for a large proportion of the total expenditure incurred in the network operation [2]. Considering the energy consumption cost from different pumps [19–21], the objective function for the operational control of water distribution networks can be formulated as

2.1 $$J(\theta )=\sum_{t=1}^{N_{T}}\left\{\sum_{i=1}^{N_{m}}C_{i,t}^{m)}W_{i,t}^{m)}(\theta_{t})\right\},$$

where *N*_*T*_ and *N*_*m*_ denote the total number of time intervals within an optimization horizon and the total number of pumps from all pumping stations, *C*^*m*)^_*i*,*t*_ (£/kWh) represents the energy tariff defining unit cost of electricity consumed by pump *i* at time interval *t*, and *W*^*m*)^_*i*,*t*_(***θ***_*t*_) (kWh) is the energy consumption of pump *i* at time interval *t* with a control solution vector ***θ***_*t*_ = [*θ*^*m*)^_1,*t*_, …, *θ*^*m*)^_*N*_*m*_,*t*_]^T^ for all the pumps (*θ*^*m*)^_*i*,*t*_ is the control setting of pump *i* at *t*). Here, the parenthesis in *m*) is used to indicate that this is an identifier of pump, so as not to be confused with exponents. Therefore, the solution vector across an optimization horizon for the whole set of operational variables can be denoted as ***θ*** = [***θ***^T^_1_, …, ***θ***^T^_*N*_*T*__]^T^. Noting if there are also other operational variables that are considered optimizable in the water network, such as the openness of valves, the solution vector at time interval *t* can be correspondingly expanded as ***θ***_*t*_ = [*θ*^*m*)^_1,*t*_, …, *θ*^*m*)^_*N*_*m*_,*t*_, *θ*^*v*)^_1,*t*_, …, *θ*^*v*)^_*N*_*v*_,*t*_]^T^, where *θ*^*v*)^_*j*,*t*_ (*j* = 1, …, *N*_*v*_) defines the *j*th valve's state at *t* and *N*_*v*_ is the total number of valves.

To calculate energy consumption *W*^*m*)^_*i*,*t*_ from every pump *i* during time interval *t*, the corresponding energy consumption rate *P*^*m*)^_*i*,*t*_ (kW) needs to be determined. It depends on several parameters, including the flow through the pump (*Q*^*m*)^_*i*,*t*_, m^3^/s), the dynamic head supplied by the pump (*H*^*m*)^_*i*,*t*_, m) and the wire-to-water efficiency (*η*^*m*)^_*i*,*t*_, %) [12], which can be expressed as

2.2 $$P_{i,t}^{m)}=\lambda \frac{Q_{i,t}^{m)}H_{i,t}^{m)}}{\eta_{i,t}^{m)}},\;i=1,\ldots ,N_{m},\;t=1,\ldots ,N_{T},$$

where *λ* is a given constant describing the specific weight of water (kN m^−3^, 9.807 under normal conditions). The product of the energy consumption rate *P*^*m*)^_*i*,*t*_ and the time parameter (i.e. time interval) in hours gives the energy consumption *W*^*m*)^_*i*,*t*_ for pump *i* at time interval *t*. In addition, it is worth mentioning that a peak demand charge of energy may sometimes be applied along with the pump operational cost. The peak demand charge is used to account for an extra utility cost (£ kW^−1^), based on the pump's highest capacity (i.e. highest energy consumption rate) operated during the billing period (measured in kW typically with a minimum of a continuous 15-min interval). Such a peak demand cost can be added into the objective function as defined in (2.1).

To minimize the objective (2.1), there are also a number of constraints to be considered for network operation. According to the nature of constraints, they can be divided into actuator constraints, hydraulic constraints and operational constraints, each being formulated as follows.

— *Actuator constraints*. Specifying the physical space of actuation variables, within which control solutions are generated. This relates to a set of permissible control inputs that can be placed upon pumps and valves in the water distribution network, which can be defined as (*t* = 1, …, *N*_*T*_):
  2.3 $$\theta_{i,t}^{m)}\in S_{i}^{m)},\theta_{j,t}^{v)}\in S_{j}^{v)},\;i=1,\ldots ,N_{m},\;j=1,\ldots ,N_{v},$$
  where pump and valve control settings, i.e. *θ*^*m*)^_*i*,*t*_ and *θ*^*v*)^_*j*,*t*_, are, respectively, constrained by set **S**^*m*)^_*i*_ for pump *i* and by **S**^*v*)^_*j*_ for valve *j*. Specifically, as for fixed-speed pumps, only the discrete switch states (i.e. on and off) is captured in **S**^*m*)^_*i*_. On the other hand, the continuously allowable speed setting is contained in this set for variable-speed pumps. Similarly, for valve settings, the allowable continuous or discrete states of the corresponding valve constitute **S**^*v*)^_*j*_.
— *Hydraulic constraints*. Specifying the hydraulic mechanism that must be followed within a water distribution network. This, in particular, refers to the conservation laws of mass and energy [1,29]:

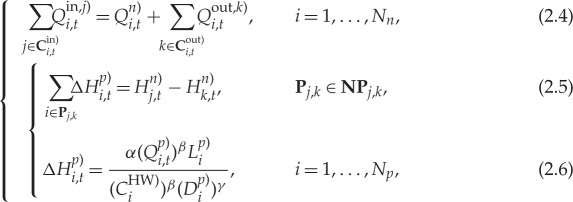

where *Q*^in, *j*)^_*i*,*t*_(m^3^ s^−1^) is the *j*th flow of water entering into node *i* at the time instant *t* (**C**^in)^_*i*,*t*_ being the set of incoming flows), *Q*^out, *k*)^_*i*,*t*_(m^3^ s^−1^) is the *k*th flow of water leaving from node *i* (**C**^out)^_*i*,*t*_ being the set of outgoing flows), *Q*^*n*)^_*i*,*t*_(m^3^ s^−1^) is the demand of water at node *i*, *H*^*n*)^_*j*,*t*_ (m) and *H*^*n*)^_*k*,*t*_ (m) are the pressure head at nodes *j* and *k*, **NP**_*j*,*k*_ are a set of paths connecting node *j* to *k*, **P**_*j*,*k*_ is a set of successively connected pipes to make up a path in **NP**_*j*,*k*_, Δ*H*^*p*)^_*i*,*t*_ (m) and *Q*^*p*)^_*i*,*t*_(m^3^ s^−1^) are the head loss and flow in pipe *i*, *L*^*p*)^_*i*_ (m), *D*^*p*)^_*i*_ (m) and *C*^HW)^_*i*_ (unitless) are the length, diameter and Hazen–Williams roughness coefficient of pipe *i*, *α*, *β* and *γ* are constant parameters, and *N*_n_ and *N*_p_ are the number of nodes and pipes in the water network. Overall, the mass conservation law (2.4) is used to describe the continuity of flow at every node in the network, while the energy conservation law defined in (2.5) and (2.6) states that the pressure head losses accumulated between two nodes should be the same for any paths connecting them. Among various hydraulic simulators available in the market, Epanet [30] is the most common one in the public domain that can be used to solve the above network hydraulic constraints.
— *Operational constraints*. Specifying the operational considerations for the running of a water distribution network. This includes minimum pressure constraints, reservoir level constraints and pump constraints [4,30]:

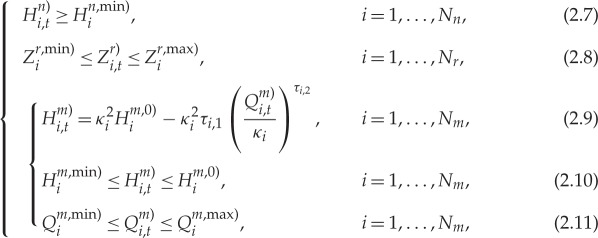

where *H*^*n*,min)^_*i*_ (m) is the minimum pressure head required for node *i*, *Z*^*r*)^_*i*,*t*_, *Z*^*r*,min)^_*i*_ (m) and *Z*^*r*,max)^_*i*_ (m) are the water level and associated operational bounds for reservoir *i*, respectively, *N*_*r*_ is the total number of reservoirs, *H*^*m*,0)^_*i*_ is the shut-off head for pump *i*, *τ*_*i*,1_ and *τ*_*i*,2_ are pump curve coefficients, *κ*_*i*_ is the pump's relative speed to its nominal speed (i.e. *κ*_*i*_ times faster than the nominal speed), *H*^*m*,min)^_*i*_ is the minimum head supplied by pump *i* corresponding to the supplied maximum flow *Q*^*m*,max)^_*i*_, and *Q*^*m*,min)^_*i*_ = 0 is the minimum supplied flow corresponding to the shut-off head *H*^*m*,0)^_*i*_. Here, in particular, a pump's characteristics are defined and confined by the pump curve, describing the relationship between the dynamic head and flow rate supplied as given in (2.9). Such a pump head decreases as flow increases and the pump heads and flows are thereby bounded by (2.10) and (2.11) given the nominal speed setting. Similar to hydraulic constraints, the pump constraints can usually be satisfied given a hydraulic simulator.

## 3. Differential evolution

DE was devised by Storn & Price [31] as a metaheuristic solver for continuous optimization problems, often exceeding other metaheuristic methods such as GAs and PSO. As in GAs, DE operates on the basis of generations of improvement of populations encoding a set of solutions for the problem of interest [32]. The population at each generation successively undergoes processes of mutation, crossover and selection, from which the individuals contained are gradually evolved to the ones giving better solutions. Each of the corresponding three operators is described as follows.

— *Mutation operator*. Denoting the *i*th individual/solution vector at the *k*th generation as ***θ***^*k*,*i*)^ (*i* = 1, …, *N*_pop_, *k* = 1, …, *N*_gen_), a trial solution ***𝜗***^*k*+1,*i*)^ for the next generation is generated using the following formula:
  3.1 $$\vartheta^{k+1,i)}=\theta^{k,i)}+F(\theta^{k,\mathrm{best})}-\theta^{k,i)})+F(\theta^{k,i1)}-\theta^{k,i2)}),$$
  where *i*1 and *i*2 are two indexes (*i*1≠*i*2, *i*1≠*i*, *i*2≠*i*) randomly generated between value 1 and the population size *N*_pop_, *F* is a scaling factor controlling the perturbation size, and ***θ***^*k*,best)^ is the best solution vector in the current (*k*th) generation of population. It is worth mentioning that the above mutation strategy is commonly referred to as DE/current-to-best/1. It has been selected from other alternatives (e.g. DE/rand/1, DE/best/1, DE/rand/2 and DE/best/2) given its better convergence properties and the ability to produce optimal solutions [31,33,34].
— *Crossover operator*. This occurs between the mutated individual ***𝜗***^*k*+1,*i*)^ and the original individual ***θ***^*k*,*i*)^ on each dimension of their included solutions. The following criterion is adopted to perform the crossover operation:
  $$\vartheta^{k+1,i,j)}=\{\begin{array}{ccc} \vartheta^{k+1,i,j)},\; & rand(\cdot )\leq CR\;\text{or}\;j=I_{j},\; & \left(3.2\right)\\ \theta^{k,i,j)}, & \text{otherwise}.\;\;\;\; & \left(3.3\right) \end{array}$$
  Here, *CR* is the crossover rate, *I*_*j*_ is a random number generated between value 1 and the number of decision variables *N*_*dv*_, *rand*( · ) produces a uniformly distributed random number in the interval [0, 1], and *𝜗*^*k*+1,*i*,*j*)^ and *θ*^*k*,*i*,*j*)^ are, respectively, the *j*th entry (*j* = 1, …, *N*_*dv*_) of solution vectors ***𝜗***^*k*+1,*i*)^ and ***θ***^*k*,*i*)^.
— *Selection operator*. This simply compares between each pair of the intermediate solution vector ***𝜗***^*k*+1,*i*)^ (obtained from operations of mutation and crossover) and the original solution vector ***θ***^*k*,*i*)^, and select the as good or better ones from the intermediate solutions to be passed onto the next generation:
  $$\theta^{k+1,i)}=\{\begin{array}{ccc} \vartheta^{k+1,i)},\; & J(\vartheta^{k+1,i)})\leq J(\theta^{k,i)}), & \;\left(3.4\right)\\ \theta^{k,i)},\; & \text{otherwise}.\;\;\;\; & \;\left(3.5\right) \end{array}$$

The general procedure for performing a DE evolution process is briefly described as follows.

*Step 1*. Generating an initial population for the problem at hand with *N*_pop_ individuals, i.e. ***Θ***^*k*)^ = [***θ***^*k*,1)^, …, ***θ***^*k*,*N*_pop_)^], *k* = 0.
*Step 2*. Calculating the fitness value of every individual in the current population.
*Step 3*. Performing mutation operation for each individual in the current population with a scaling factor *F*.
*Step 4*. Performing crossover operation for each individual in the current population with a crossover rate *CR*.
*Step 5*. Calculating the fitness value of every new individual resulting from mutation and crossover.
*Step 6*. Performing selection operation to generate a new population for the next generation.
*Step 7*. *k* = *k* + 1, moving to step 3 for next generation's evolution. Continuing the procedure until some stopping criterion is satisfied, e.g. the maximum number of generations (*N*_gen_) is reached.
*Step 8*. Output ***Θ***^*k*)^ and the best solution to the problem.

## 4. The methodology for water network operational optimization

Given the preliminaries of the water network operation and corresponding problem formulation as presented in §2, this section presents the systematic optimization methodology for realizing real-time operation, including the control scheme, encoding of network variables and the mixed-integer DE paradigm.

### (a) Model-based predictive control scheme

**Figure 1.**
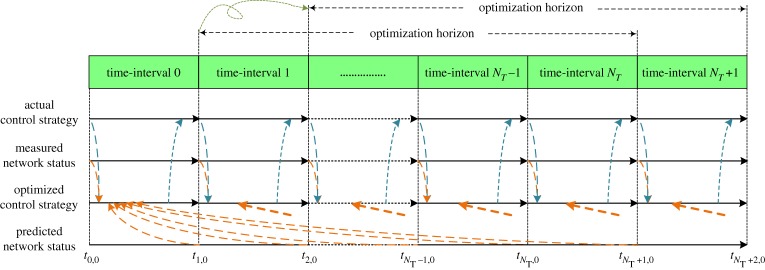
Water network model-based predictive control sequence diagram. The actual control strategy together with the network status at the beginning of every time interval is transmitted from the field to the controller. The controller then performs operational optimization based on the input of this information and the predictions of network statuses, after which the control strategy is computed and sent back to the field for actuation. The entire optimization horizon is dynamically moved one step ahead upon the arrival of a new time interval. (Online version in colour.)

Model-based predictive control as an advanced control concept has been gradually adopted in many domains such as sewerage system control [35,36]. In this paper, the clean water network operational optimization employs a model-based predictive control scheme, the corresponding control sequence diagram being illustrated in figure 1. The operation of clean water networks is established on the continuously rolling optimization horizon, consisting of a number of time intervals determined by the network sensing infrastructure (e.g. 1 h). Assuming that we are now just entering time interval 0 as shown in figure 1, the aim is to find out the next time-step's control signal for pumps and valves at time instant *t*_1,0_, while minimizing the objective defined in (2.1) and meeting constraints (2.3)–((2.11)). In order to do so, the control strategies of pumps and valves including those optimizable and non-optimizable, together with the statuses of other monitored network variables (e.g. service reservoir levels) at the start (i.e. *t*_0,0_) of time interval 0 are first gathered from the field and sent for optimization. The network statuses at the end of this time interval are thereby predicted through enquiring the network hydraulic model based on the collected field control settings and network statuses.

Such predicted network statuses are then set as the initial point to perform a series of length of *N*_*T*_ extended-period network simulations, each along with a set of trial control strategies across the forthcoming optimization horizon. Accordingly, the predicted network statuses for the next *N*_*T*_ time intervals upon the use of these control strategies are successively derived for assessment. Here, the control strategies are designated and optimized by the mixed-integer DE algorithm to be devised in the following subsections. As a result of the network operational optimization, the optimized control strategies from time interval 1 to *N*_*T*_ can be transmitted back to the field for actuation. However, it is envisaged that only the optimized strategies at the next time-step are going to be actuated as the remaining strategies will be updated once a new set of field monitoring states are made available. Therefore, for simplicity, only the control strategies of the next time-step are transmitted for actuation as illustrated in figure 1, though those from the whole optimization horizon are obtained.

The above control process repeats at every time interval, thus providing real-time solutions to the network operational problem in response to the dynamic change of the field. As in time interval 0, the same process runs for time interval 1, …, *N*_*T*_, *N*_*T*_ + 1 as in figure 1. For simplicity, the predicted network statuses therein across different time intervals are instead denoted as a bold dashed line. It is worth mentioning that in the circumstance of communication anomaly, the rest of the control strategies (at time-steps other than the next one) stored in field controllers can be adopted for temporary operation of the water network. It can be seen that the total time available for deriving network control strategies, including gathering and processing of field monitoring data, the conduction of the optimization task and the transmission of the control strategy back to the field, should be limited within one control time interval. Moreover, the actual control strategies are usually the same as the optimized ones given that the optimized solutions are eventually actuated. Nevertheless, the field engineers or network controllers are obviously allowed to intervene at any point in the network control process. In this case, the actual control strategies are unknown and thus need to be monitored together with other field variables. The optimization to be conducted will then be able to search for new solutions given such changes.

### (b) Encoding strategy

As the standard DE was designed for continuous optimization problems, it cannot be directly applied to the case where discrete decision variables are involved. A general straightforward method is to use the mapping techniques, such as rounding-off approaches, where the continuous values are transformed into the proper discrete values (e.g. integers) for consideration. The use of such rounding-off strategies can reduce the algorithm performance and solution optimality, partially due to the limited ability to efficiently explore the search space (where redundancy is introduced) and fully comply with various algorithm operations and settings, as in dealing with continuous variables [37–39]. Besides, a high degree of nonlinearity is exhibited by the water network operational optimization problem, given the combinatorial effect of the hydraulic complexities, operational constraints, demand profiles, energy tariffs and, both discrete and continuous operational variables. To efficiently explore the search space and apply the algorithm operations, in this paper, a mixed-integer DE algorithm is devised to directly handle the discrete-continuous actuation variables (through inherent encoding of variables and precise control of variable operations) involved in the water network for energy cost minimization, while considering various physical, hydraulic and operational constraints all together.

The encoding of decision variables (i.e. variables that are optimizable) contained in the water network is now described to transform the operational problem into a form that can be processed by the optimization algorithm. The decision variables are determined with the input from network operators taking into account any security and operational concerns. Here, in particular, the decision variables refer to the actuators (i) that can be physically and remotely controlled and monitored and (ii) that are allowed to be automatically optimized from the operator's perspective without impairing their key business requirements. All the pumps and valves presented in the network should be investigated to distinguish whether they are optimizable or not. Furthermore, according to the continuity of their solution space, they are differentiated as discrete and continuous actuation variables. For example, a fixed-speed pump has only two discrete states (i.e. on/off), while a variable-speed pump can have either several configured discrete states or any continuously varying states (bounded or not). Similarly, for valves, they can also be differentiated as discrete (i.e. closed and opened, or several adjustable active settings) and continuous (i.e. any active settings bounded or not) variables. As a result, this leads to a mixed-integer operational optimization problem given the involvement of both discrete and continuous decision variables.

**Figure 2.**
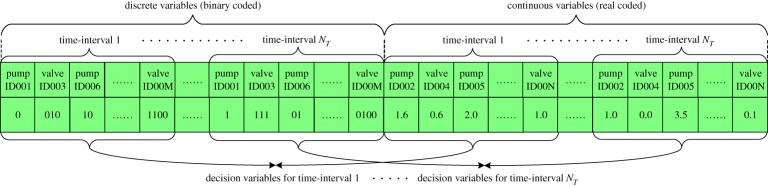
Encoding of decision variables in the water network. (Online version in colour.)

The encoding of all the decision variables is illustrated in figure 2. For a given time interval (1, …, *N*_*T*_), the states of optimizable pumps and valves are coded in either binary strings (for discrete variables) or real values (for continuous variables). The encoding is then repeated for each time interval within an optimization horizon to form the whole set of decision variables to be optimized. In detail, for pumps and valves with discrete actuation states, they are coded by means of several bits as a string representing the chosen actuation from their permissible control inputs. For example, a valve with four controllable settings would need a two-bit string to be coded, while six settings require a three-bit string. On the other hand, for pumps and valves with continuously controllable states, real-value encoding is used to indicate the underlying setting being chosen. In the end, as shown in figure 2, for every time interval, the decision variables from the discrete part plus the ones from the continuous part constitute the control strategies for pump and valve operation.

### (c) Mixed-integer differential evolution algorithm

Based on the encoding of decision variables presented in the previous subsection, this subsection introduces the mixed-integer DE algorithm proposed within our methodology. According to the description of preliminaries given in §2, the optimization of clean water network operation can be generally defined as follows:

4.1 MinimizeJ(θ),subject to(2.2)–(2.11).

Of these constraints, (2.2) is employed to compute energy consumption based on the fulfilment of constraints (2.4)–(2.6) and (2.9)–(2.11), using the developed hydraulic model for the network of interest. Thus, these equality-related constraints are implicitly satisfied given the hydraulic model simulation. Then, constraint (2.3) describes the continuous/discrete nature of the network actuation variables expressed by the encoding strategy. It will be continuously met during the optimization process through a later designed strategy for actuation constraint satisfaction. The remaining operational constraints (2.7) and (2.8), are the inequality constraints required to be explicitly integrated into the optimization process, which can be first transformed as follows:

4.2 $$g_{i}(\theta )\leq 0,\;i=1,\ldots ,N_{ie},$$

where *g*_*i*_(***θ***) denotes the *i*th converted inequality constraints (after normalization) and there is a total of *N*_*ie*_ such converted constraints. Correspondingly, to assess the fulfilment level of constraints, the degree of constraint violation is given as follows:

4.3 $$J_{i}(\theta )=\max\{0,g_{i}(\theta )\},\;i=1,\ldots ,N_{ie}.$$

Based on the above formulation, DE is adopted as the metaheuristic algorithm to optimize water network operation. As DE was originally devised for continuous variable optimization, its modified and tailored version for such a constrained mixed-integer optimization problem is now given as follows.

1. Initial solution generation. To improve optimization efficiency, the initial solutions for the water network operational optimization are generated jointly using three different strategies. First, by simulating the water network with existing operating rules (upon the current network statuses) for the whole optimization horizon, the initial solutions are seeded around the tried-and-tested existing strategies. Then, the previously optimized solution including control strategies between time interval 2 and *N*_*T*_ and its variants are also employed to generate the initial solutions from time interval 1 to *N*_*T*_ − 1. Finally, the rest of the initial solutions are randomly generated satisfying the actuator constraints in order to sustain the diversity of the population.

2. Mutation operation. For binary strings shown in figure 2, it follows

4.4 $$\vartheta_{b}^{k+1,i)}=\theta_{b}^{k,best)}\;⊕\;\left(\theta_{b}^{k,i1)}\;⊕\;\theta_{b}^{k,i2)}\right),$$

where ***𝜗***^*k*+1,*i*)^_*b*_, ***θ***^*k*,*best*)^_*b*_, ***θ***^*k*,*i*1)^_*b*_ and ***θ***^*k*,*i*2)^_*b*_ are binary strings from the *i*th mutated vector, and the best, *i*1)th and *i*2)th original vectors, respectively. Sign ⊕ is the logical exclusive (XOR) operation. The algorithm can thus search the discrete space defined by the objective function and constraints by updating multiple trajectories of solutions. The move of each solution is thereby guided by a deterministic element (towards the best solution) and a stochastic element (introduced by the two randomly selected solutions). This trajectory updating scheme is designed to balance the exploration and exploitation capability in searching for optimal solutions. As for continuous variables, they are mutated in the same way as in a standard DE algorithm given in §3.

3. Crossover operation. For binary strings shown in figure 2, its crossover operation can be adapted from the one for continuous variables:

$$\vartheta_{b}^{k+1,i,j)}=\;\{\begin{array}{ccc} \vartheta_{b}^{k+1,i,j)}, & rand(\cdot )\leq CR\text{ or }j=I_{b,j}, & \;(4.5)\\ \theta_{b}^{k,i,j)}, & \text{otherwise}, & \;(4.6) \end{array}$$

where *𝜗*^*k*+1,*i*,*j*)^_*b*_ and *θ*^*k*,*i*,*j*)^_*b*_ are, respectively, the *j*th entry (*j* = 1, …, *N*_*b*_) from solution vectors ***𝜗***^*k*+1,*i*)^_*b*_ and ***θ***^*k*,*i*)^_*b*_ (*N*_*b*_ being the length of binary strings), *I*_*b*,*j*_ is a random number generated between 1 and *N*_*b*_. For continuous variables, they are operated the same as in a standard DE.

4. Actuator constraint satisfaction. For the *j*th binary-coded discrete control variable, the corresponding set of binaries are decoded such that the obtained value directly meet its constraint:

4.7 $$\vartheta_{d}^{k+1,i,j)}=\frac{\theta_{d,\max}^{k+1,i,j)}\vartheta_{d}^{k+1,i,j)}}{\vartheta_{d,\max}^{k+1,i,j)}}-\frac{\vartheta_{d}^{k+1,i,j)}}{\vartheta_{d,\max}^{k+1,i,j)}}+1,$$

where $\vartheta_{d,\max}^{k+1,i,j)}$ denotes the maximum positive value that can be represented by the number of binaries coded, $\theta_{d,\max}^{k+1,i,j)}$ is the total number of admissible choices for the *j*th discrete variable (*j* = 1, …, *N*_*d*_), and *N*_*d*_ is the number of discrete variables. The integer value *𝜗*^*k*+1,*i*,*j*)^_*d*_ decoded from binaries, on the right-hand side of (4.7), can then be transformed and rounded to the nearest integer corresponding to the choice for the *j*th discrete variable under consideration. On the other hand, as for real-coded continuous variables, the values are simply examined to be directly restricted within the required ranges.

5. Fitness evaluation. Both the objective and constraints are assessed using the following penalty-based constraint handling method:

4.8 $$\mathrm{Fitness}=J(\theta )+\varpi \sum_{i=1}^{N_{ie}}J_{i}(\theta ),$$

where *ϖ* is the trade-off parameter controlling the balance between objective minimization and constraint satisfaction. The purpose of employing the above method is to reduce the number of adjustable parameters (given the normalization of different constraints), while considering feasible and infeasible solutions simultaneously in the optimization process in order to maintain the diversity of solutions. As a consequence, the infeasible solutions are not only assessed by their degrees of violation of constraints, but also examined according to their objective values produced. A simple example is given here to illustrate the benefit. Thinking of two infeasible solutions, first of which has a smaller degree of constraint violation (e.g. 2.0) but with a very large objective value (e.g. 1000.0), while the other possessing a slightly larger constraint violation (e.g. 2.2) together with a very small objective value (e.g. 10.0). It is straightforward to choose the second solution as it gives considerably better optimized objective function at not much additional expense on the constraint violation. This is particularly useful when the optimization process is first initiated and most solutions are infeasible and associated with objective values in different orders of magnitude, where it is essential to quickly locate some acceptable infeasible solutions with small objective values. One thing to bear in mind, for certain scenarios, the optimization may not be always able to search for a feasible solution; then, finding an infeasible solution with minimized objective value as well as acceptable constraint violation is of particular importance.

6. Selection and termination. This performs the same as in a standard DE algorithm. Replace the individuals with the correspondingly evolved better ones. Then, go to step 2 if the optimization continues; otherwise, stop the optimization process.

## 5. Experiments

In this section, the effectiveness of the proposed methodology for water network operational optimization is demonstrated with the aid of a hydraulic model constructed from a real pilot network. Following the design of the proposed optimization methodology, it was then implemented in C with the integration of Epanet and metaheuristics libraries. Here, a network input file including the physical water network descriptions and configurations was generated as a result of the hydraulic model development. Then, the Epanet library together with the network input file was integrated into the optimization process to perform network simulations under different operational strategies. On the other hand, the environment provided by the Genetic Algorithm Utility Library (Gaul) [40] was employed as the basis to develop the proposed constrained mixed-integer DE algorithm. For comparison purposes, the well-known Deb's single-objective GA algorithm in C (Real + Binary + Constraint Handling) [41] was also implemented within the proposed optimization methodology. To allow flexibility, a C library file realizing the proposed methodology as a whole was thereby produced to provide the service of water network operational control, where a set of APIs (application program interfaces) are associated for programmatic access.

### (a) Pilot area description

The pilot area is a water distribution zone situated in the rural area of the west coast of North Wales. It is a wholly enclosed system from abstraction, through to water treatment and distribution. There are two raw water abstraction points, where two individual raw water mains carry the raw water to the water treatment works (WTW) at Village A. The treated water is then transported to Village C via Village B. The reasons for choosing this site as the pilot area were mainly attributed to its several unique characteristics, such as self-contained network, water deficit zone and highly volatile demand.

**Figure 3.**
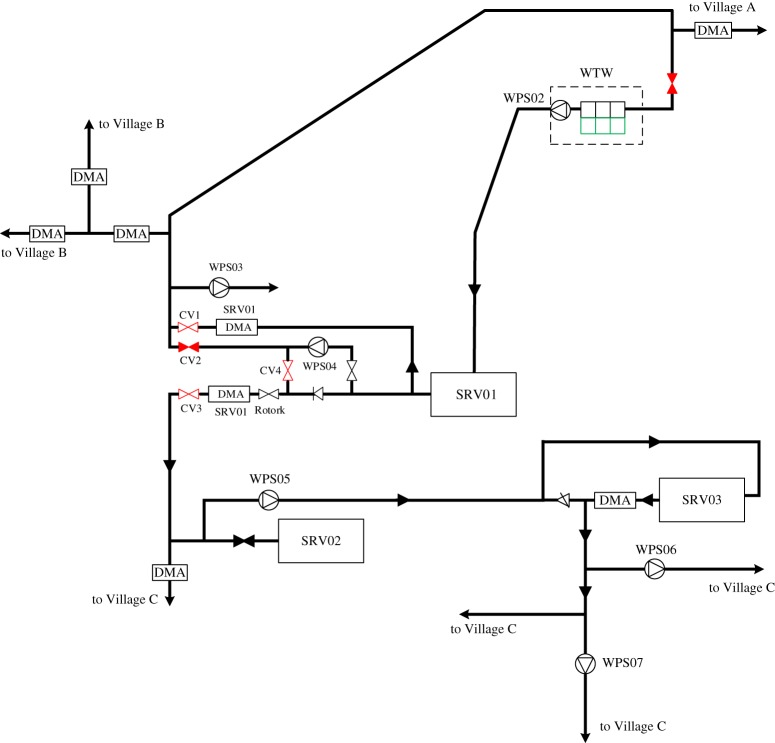
Schematic of the pilot water distribution network in the west coast of North Wales. (Online version in colour.)

Figure 3 depicts the skeletal schematic of the water distribution network within the pilot area. As the information about the actual assets in the network is either commercially sensitive or is information relating to critical infrastructure, their names have been anonymized. As shown in the network schematic, the final water obtained from the only WTW on the top right corner of the figure is first gravitated to SRV01 (service reservoir), where WPS02 (water pumping station) is for pressurizing water within Village A. There is one working pump within each pumping station in this pilot network. At this point, it should be noted that there is a small final water tank in the WTW where the treated water is temporarily stored before leaving to SRV01. At the outlet from the tank, the final water is sampled for free and total chlorine, aluminium, turbidity, colour, pH and conductivity; a sample also goes to the Cryptosporidium unit. Originally, there was an extra pumping station WPS01 delivering water to farms that has been deprecated around the WTW.

The SRV01 is a twin compartment reservoir located in Village B, where the final water enters and leaves through two outlet mains. The two mains enter a valve house where the final water is sampled for free chlorine and pH with a separate line feeding the final water sample tap. After sampling the final water passes into distribution mains supplying Villages C, B and A. It can be seen that a booster pump WPS04 is installed on a cross connection between the mains to boost the pressure into the Village C main when required. If water level at SRV02 drops to 2.5 m, the pump comes on and stays on until level reaches 2.9 metres. In addition, when water level at SRV02 reaches 3.2 m, a signal is sent to shut the Rotork (valve) around SRV01. When water level drops to 2.93 m, the Rotork opens fully. In Village B, WPS03 is used to boost the pressure to supply a small number of properties. There are a number of DMAs (district metering areas) that exist to facilitate the water management process within Village B.

In Village C, SRV02 is the first reservoir used for storing and providing pressurized potable water for the area. It has a combined inlet/outlet main which gravitates to the lower area of Village C and WPS05. WPS05 then pumps water (via a 160 mm HPPE water main) to the second reservoir in the area, i.e. SRV03, and is controlled according to the inside water level (reservoir's inlet), where level below 2.22 pumps on and level above 2.57 pumps off. The outlet main of SRV03 then gravitates to the lower area of Village C, WPS06 and WPS07. These two pumping stations (WPS06 and WPS07) are for supplying water to customers resident in high elevations. Finally, it is worth mentioning that a minimum nodal pressure head of 20 m is required for supplying water in the whole pilot area.

**Figure 4.**
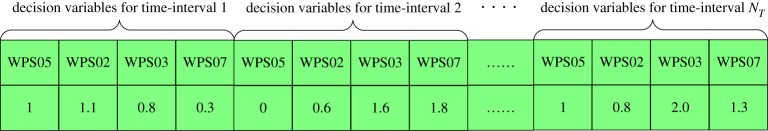
Solution representation for the operation of the pilot water network. (Online version in colour.)

Except for WPS04 and WPS06 that are fixed speed pumps, the rest are variable speed pumps. Regarding the energy tariff, WPS02 and WPS07 adopt an adaptive pricing scheme, while the rest have a flat price across the day, all also incurring additional charges such as feed in tariff, admin cost and renewable charge. Given the existing control rules, pumps WPS02, WPS03, WPS06 and WPS07 are always switched on at fixed speeds to provide water to single properties, while WPS04 and WPS05 are level controlled according to the water levels in SRV02 and SRV03, respectively. With the aid of the Rotork controlling water entering into SRV02, WPS04 is rarely switched on thus not to be optimized. Water levels in SRV01 are simply regulated by an upstream valve. Owing to their nature, WPS02, WPS03 and WPS07 are optimized as variable-speed pumps. In addition, WPS05 must be running at its maximum speed to deliver water to SRV03 at high elevation, thus it is optimized as fixed-speed pumps. WPS06 is non-optimizable as it is connected to single properties while also fixed-speed, thus having to be switched on all the time. Originally, there is one critical node identified by the water network operator and two operational ranges related to SRV02 and SRV03. As SRV01 is well regulated given the current practice thus there is no operational range in existence. Furthermore, three demand nodes are additionally identified as the critical nodes in response to the three optimizable variable-speed pumps in order to supply water meeting minimum pressure requirements. It is worth highlighting that to achieve better optimization performance, we hereby choose to exclude WPS04 from optimization as (i) it is found that WPS04 is never required to be operational, (ii) with the optimization, it could still be turned on occasionally as WPS04 is a very small pump and consumes little energy compared to others (thus posing little impact on the optimization) and (iii) the increased optimization burden resulting from both current and future decision variables being considered within an optimization horizon, and the involvement of various physical, operational and hydraulic constraints as well as time considerations. Therefore, such heuristic knowledge is thus encouraged to be used in combination with the optimization, while also saving optimization time given this real-time operational problem. As a result, the final solution representation for the pilot network is illustrated in figure 4.

Regarding the size and complexity of the problem, it can be discussed from two related perspectives: i.e. the size of the pilot water network being dealt with and the complexity of the optimization problem resulting from the pilot water network and optimization methodology. On the one hand, the pilot network delivers potable water over 10 km distance and contains 1831 junctions, 1397 pipes, six pumping stations and three service reservoirs, with a maximum capacity of 2.6 megalitres d^−1^ (where a person approximately consumes 150 l of water per day in the UK [42]). It is a fairly small but representative network for supplying water in an area including several local villages, while providing realistic applications compared to the commonly seen benchmarks (e.g. Anytown and New York City Tunnel networks, where dozens of nodes and pipes are considered [1]). On the other hand, as shown in figure 4, the resulting optimization problem from the pilot network contains 48 discrete-continuous decision variables considering an optimization horizon of 12 h and four pumping stations, and this optimization repeats at every time interval according to the model-based predictive control scheme. For the underlying pilot network, as shown later in table 2, the optimization took around 10 minutes to produce the control strategy at each time interval (well below the 1 h time-interval allowance). By further increasing such optimization time and possibly also exploiting high performance computing techniques (e.g. super-computing or parallel computing), it is thus anticipated that the methodology developed in the paper can be applied to similar and larger water networks.

**Figure 5.**
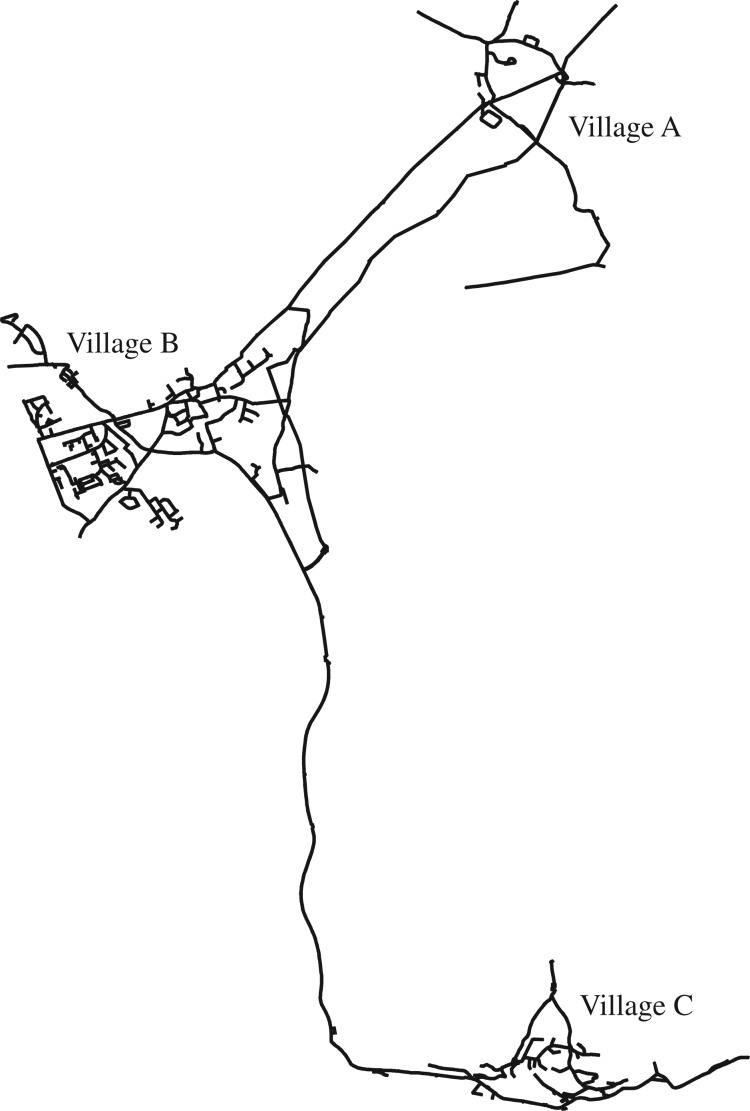
The overall layout of the pilot water network from Epanet.

To allow the assessment of network operational strategies, a hydraulic model as shown in figure 5 for this site was developed and calibrated by Welsh Water Modelling Team, including the network structure and characteristics and consumption profiles. The developed hydraulic model contains the DMAs and any trunk main back to a source such as a service reservoir. Both flow and pressure calibrations were carried out to let all flows and pressures match any logged data relevant to the area being modelled. Originally, the model was developed using SynerGEE UI. As SynerGEE did not provide a library file and the corresponding programmer's toolkit for integration with optimization, a conversion into the well-known public-domain Epanet model was carried out. Here, it is also noted that the original Epanet software only takes pump efficiency versus flow at nominal speed condition, while this can be inaccurate for other speed settings in the use of variable-speed pumps. A scaling modification to the reduced/increased flows introduced in [43] was adopted for computing more reliable power consumption rates with updated pump efficiency curves other than at the nominal speed.

### (b) Results and analysis

**Figure 6.**
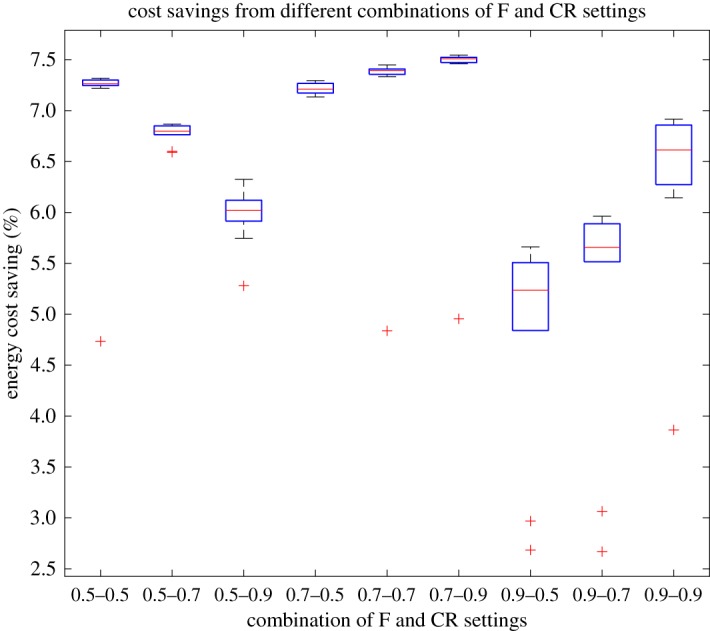
Energy cost savings from initial trials of different combinations of *F* and *CR* values in the proposed method. (Online version in colour.)

With the experimental trials, a population size of 40 and maximum generation number of 200 were used for both DE and GA optimizer [33,44,45]. In GA, the mutation rate was given as 0.09 and 0.10 for binary and real coded variables, respectively, while the crossover rate being 0.7 [41]. To see the effects of the *F* and *CR* settings in our approach and according to [31,33], a number of initial trials were performed to simulate water network operational optimization for a week under a set of different *F* and *CR* values, each repeated with ten runs (resulting in a total of 90 runs). In other words, such short trials were employed to determine their values for use in the following full simulation. The number of water network evaluations for each run of a trial setting is thus 40 × 200 × 24 × 7 = 1 344 000. The results over multiple runs for each setting are listed in table 1. It can be found that the setting of *F* = 0.7 and *CR* = 0.9 produced the best (and the best average) cost savings among others, and thus adopted in the following simulation. This can be also reflected in figure 6, where alternative box plots of results over the multiple trials are given. It is worth mentioning that given the periodic nature of this operational optimization problem (i.e. the optimization task is repeated at every time interval), the simulation of network operation over time consists of multiple runs of an optimization task in the traditional sense. Apart from the communication and basic data processing time, the optimization is allowed to make use of the majority of time defined by a control time interval, though the optimization time consumed is shortened given the complexity of this case study.

**Table 1.** Initial trial of cost savings for the different parameter settings of the proposed mixed-integer DE method

*F*	0.5	0.5	0.5	0.7	0.7	0.7	0.9	0.9	0.9
*CR*	0.5	0.7	0.9	0.5	0.7	0.9	0.5	0.7	0.9
	(%)	(%)	(%)	(%)	(%)	(%)	(%)	(%)	(%)
best	7.32	6.87	6.32	7.29	7.45	7.55	5.66	5.96	6.92
ave.	7.02	6.77	5.97	7.22	7.14	7.25	4.81	5.17	6.33
STD	0.80	0.10	0.29	0.05	0.81	0.81	1.07	1.23	0.91

After the above initial trials and given the proposed methodology, the optimization of the water network operation was then simulated from 30 April 2016 to 30 January 2017. With the original energy tariff being used, it is found that a reduction of energy expenses of 5.39% can be achieved by the modified DE paradigm compared with using the existing control rules. On the other hand, if GA is adopted within the proposed methodology, a considerably less cost reduction of 2.23% is thereby anticipated (i.e. less than half of 5.39%), demonstrating the superiority of the modified DE method designed to cope with this type of mixed-integer operational optimization problem. In addition, the average optimization time and the average degree of constraint violation for every control interval are also given in table 2. The energy cost reduction given by optimization approaches is computed as a percentage based on the existing control rules. The time parameter is not given for the use of existing control rules, as the control signal is directly adjusted according to reservoir levels and without the involvement of optimization. In comparison with the GA, it is found that our method is able to produce more energy cost savings with less constraint violations, while also consuming less computational time to generate control strategies. It is also worth mentioning that the use of such amount of time (around 10 min in this case study) sits well within the 1-h time interval, allowing further space for improved performance if the optimization time is increased in practice and for application of the methodology to larger water networks where more optimization time is needed.

**Table 2.** Simulation results of various control approaches for both the original and adaptive pricing schemes

energy tariff	approach	avg. time (s)	cost reduction (%)	avg. constraint violation
original	rule	—	—	3.09 × 10^−3^
	GA	641.01	2.23	7.69 × 10^−4^
	modified DE	591.93	5.39	5.58 × 10^−5^
adaptive	rule	—	—	3.09 × 10^−3^
	GA	640.75	18.76	3.93 × 10^−3^
	modified DE	591.85	23.69	3.61 × 10^−3^

**Figure 7.**
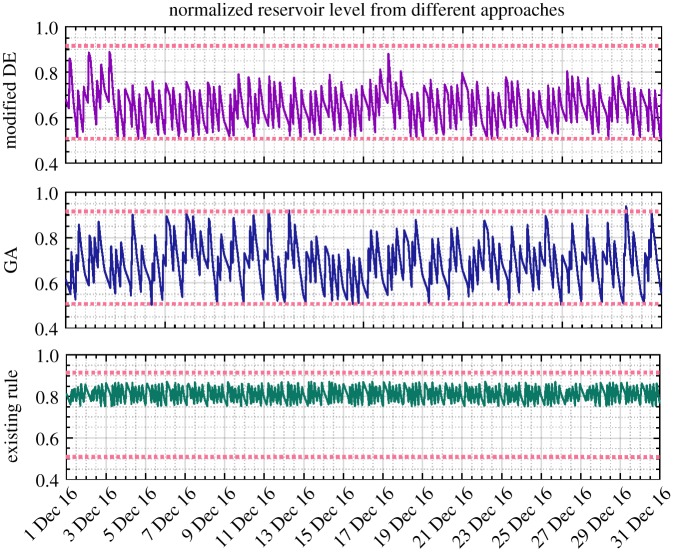
Service reservoir level (normalized, December 2016) obtained from DE, GA and existing control rules based on the original pricing scheme (the solid line denotes the corresponding values and the dotted line depicts the operational requirements). (Online version in colour.)

**Figure 8.**
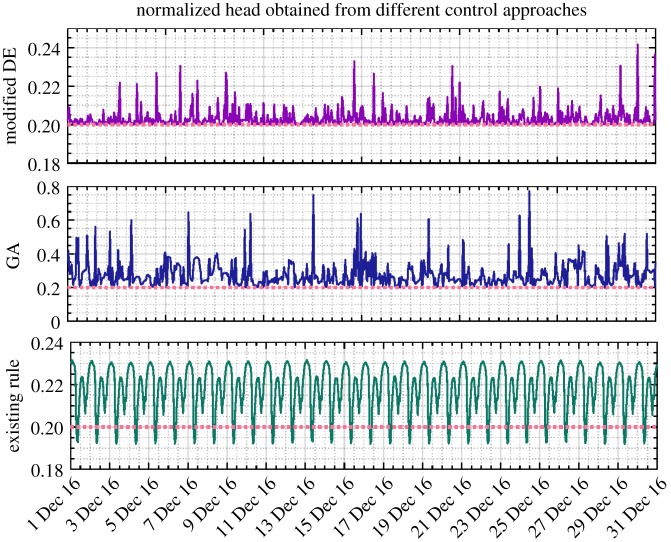
Nodal head (normalized, December 2016) obtained from DE, GA and existing control rules based on the original pricing scheme. Noting that the plots are over differing ranges to allow details to be shown. (Online version in colour.)

As an example, the reservoir levels and nodal heads (after normalization) for the last month of 2016 resulting from the modified DE, GA and existing control rules are, respectively, presented in figures 7 and 8. All the values are well constrained within the anticipated operational ranges depicted by the upper and lower dotted lines, except the slightly less pressure head than required that is occasionally provided under the existing control scheme. The exactly averaged degree of violation of constraints resulting from different approaches across the entire simulation period can be found in table 2. It should be noted that in line with the water utility's confidentiality policy, all the values presented in these two and the following figures of the paper were therefore scaled between 0 and 1. In particular, compared with the modified DE approach, the pressure head obtained by GA is unnecessarily higher, which potentially indicates suboptimal solutions being generated and thus increased energy cost.

Though the modified DE is able to reduce the energy cost by a certain degree for real-time operation of the water network, an additional step was then carried out to further investigate whether there is still space to lessen such cost given the current network configurations. As the original energy tariff used for most pumping stations (which are in turn responsible for the major energy cost) is flat (i.e. fixed pricing scheme was adopted), while only two pumping stations employ adaptive pricing, it is thus envisaged that the cost saving obtained above should be able to be significantly increased if all pumps in the network adopt the adaptive pricing scheme, thereby allowing the optimization procedure to fully leverage the complexity posed by the energy tariff. For this purpose, the proposed methodology was then continued to be examined with the adaptive pricing applied to all the pumping stations. As a result, it implies that a 21.55% reduction in energy cost can be obtained by switching to an adaptive energy tariff, while using the same existing rule to operate the water network. Importantly, it is then also found that a cost reduction of 23.69% can be further expected using our modified DE in comparison with the existing rule, both based on the full adaptive pricing scheme. In other words, this amounts to a total of 40.13% cost reduction as a result of the adoption of the modified DE optimization and full adaptive pricing, instead of the existing rule and original pricing.

**Figure 9.**
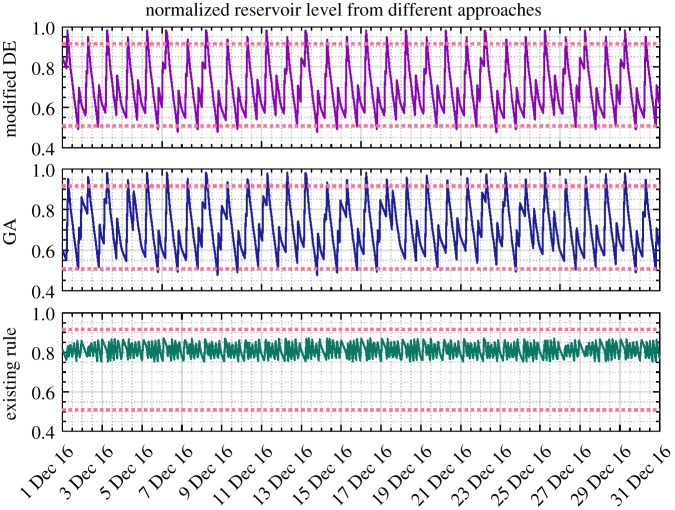
Service reservoir level (normalized, December 2016) obtained from DE, GA and existing control rules based on the full adaptive pricing scheme. (Online version in colour.)

**Figure 10.**
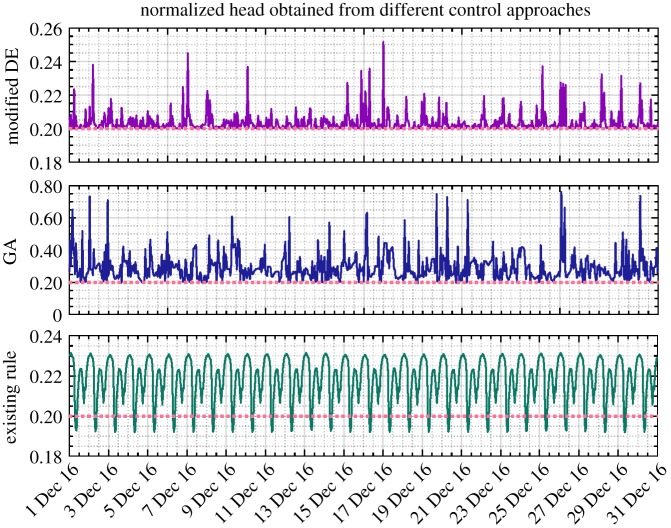
Nodal head (normalized, December 2016) obtained from DE, GA and existing control rules based on the full adaptive pricing scheme. Noting that the plots are over differing ranges to allow details to be shown. (Online version in colour.)

**Figure 11.**
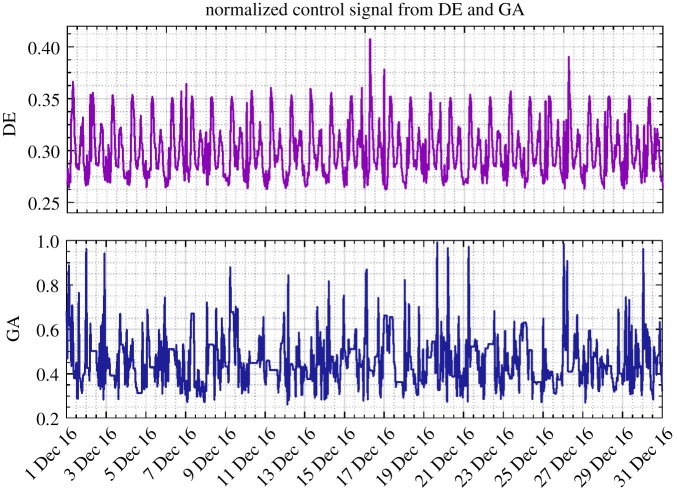
Pump control signal (normalized, December 2016) obtained from DE and GA based on the full adaptive pricing scheme. The existing control signal is fixed at a value of 0.33. Noting that the plots are over differing ranges to allow details to be shown. (Online version in colour.)

**Figure 12.**
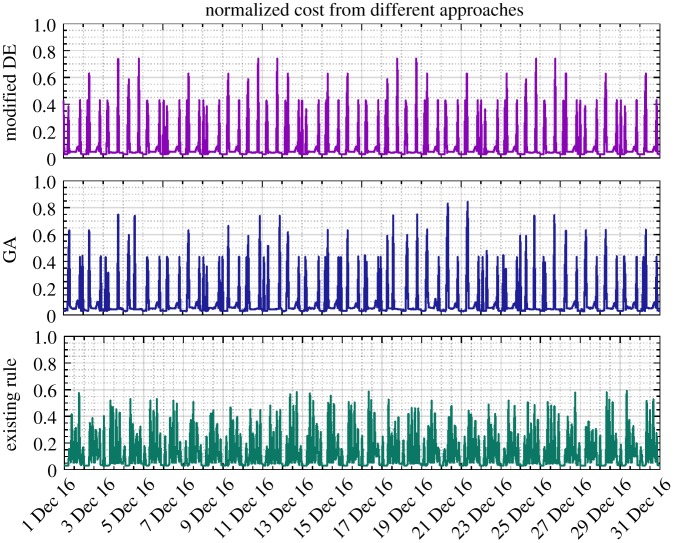
Energy cost (normalized, December 2016) obtained from DE, GA and existing control rules based on the full adaptive pricing scheme. (Online version in colour.)

In addition, as shown in table 2, given the proposed methodology applied to the scenario of full adaptive pricing, the modified DE is again confirmed to generate more savings than the GA (23.69% versus 18.76%). Meanwhile, the computational efficiency is also demonstrated therein. The resulting reservoir level, nodal head, pump control signal and energy cost from different approaches are, respectively, depicted in figures 9–12. As for level and head variables, most resulting values are well confined within the corresponding requirements, except for some slight overshoots/undershoots. The slight violation of these soft operational constraints is acceptable in practice and will not cause problems, while the degree of constraint violation in every time interval for different approaches is given in table 2. The existing control rule can also cause such violation (e.g. the minimum head violation as explicitly shown in figure 8). Notably, as shown in figure 9, unlike the narrowly distributed reservoir levels resulting from the existing control scheme, the optimized reservoir levels obtained by GA and DE are just distributed around their operational range requirement, as the optimization procedure tends to fully and timely use the capacity of the reservoir to accommodate the complexity of the varied pricing structure defined by the energy tariff. Given the optimization approach involved, in general, more energy consumption can therefore be adapted to the low pricing period of the energy tariff. In addition, compared with the modified DE, GA is again found to produce excessive nodal pressure as illustrated in figure 10. On a different note, as the existing control scheme does not factor in the energy tariff in controlling the water network, no changes are therefore seen in the resulting statuses of network variables after using the full adaptive pricing scheme.

**Figure 13.**
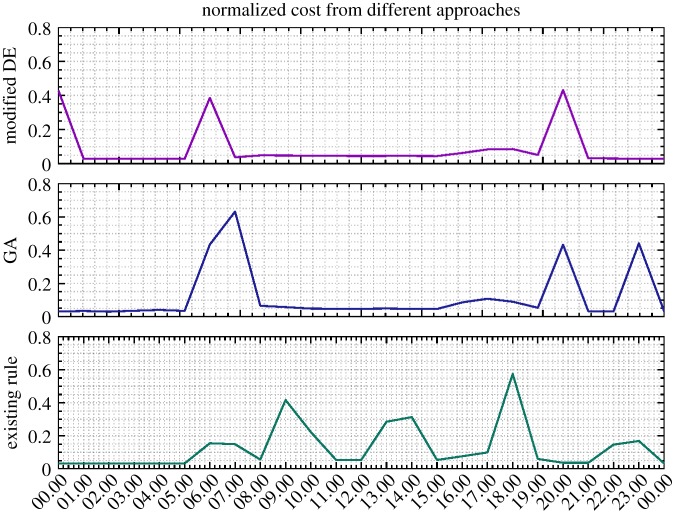
Hourly zoomed-in view of energy cost (1 December 2016) from DE, GA and existing control rules based on the full adaptive pricing scheme. (Online version in colour.)

**Figure 14.**
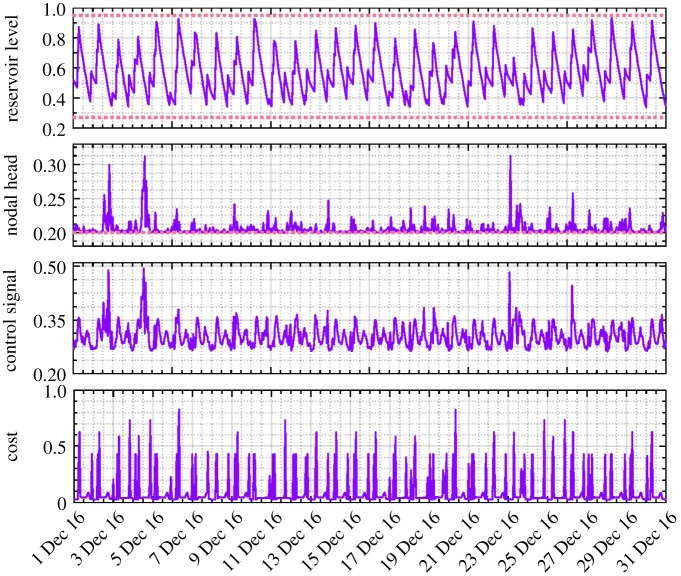
Optimized results (normalized, December 2016) obtained from the modified DE based on the full adaptive pricing scheme and relaxed reservoir operational levels. (Online version in colour.)

Moreover, as seen from figure 11, the pump speed given by GA is considerably higher than that given by the modified DE and the existing control scheme, resulting in the unnecessarily high nodal pressure and increased energy cost. Interestingly, as demonstrated in figure 12, in comparison with the existing control rule, the number of relatively high energy costs (e.g. those larger than 0.2) incurred from GA and DE is less and they are more scatteredly distributed, owing to the tariff complexity being taken into account. This can also be clearly demonstrated by the enlarged view of hourly energy costs depicted in figure 13, where relatively significant energy expenses are generated during the daytime (associated with high pricing) for the existing control rule. Furthermore, a relaxed operational range of the reservoir currently operated by Welsh Water was also examined with the modified DE method, the obtained optimization results together with the relaxed range being illustrated in figure 14. Compared with the ones depicted in figure 9, the resulting reservoir levels herein are better constrained due to the increased allowable water storage capacity. The original reservoir operational levels used in combination with the existing control rule are thereby considered more conservative. In conclusion, the proposed methodology is able to perform real-time strategies for water network operation, while reducing energy costs and taking into account a variety of constraints. The proposed mixed-integer DE algorithm is demonstrated more effective than GAs in terms of gaining more savings. It is also suggested that adaptive pricing rather than fixed pricing should be adopted in order to achieve more cost savings for the water network operation, especially when optimization techniques are introduced.

## 6. Conclusion

This paper has proposed and developed a systematic discrete-continuous optimization methodology for water network real-time operation, where a wide range of connected multidimensional factors are intrinsically considered as a whole, including the physical structure and characteristics of the network, operational requirements, water consumption profiles and the structure of energy tariff. The network operational strategy is updated dynamically according to the control scheme with little human involvement, while having the flexibility of configuring optimizable actuators and operational constraints. An adapted DE algorithm was also proposed to deal with the mixed-integer and constrained nature of the network operational optimization problem. To improve efficiency and accelerate the optimization process, an initial solution generating scheme was devised with solutions taken from existing operating rules, previous optimization and randomization. A converted hydraulic model (from SynerGEE to Epanet) for a pilot area in the U.K. was also integrated into the optimization process to achieve real-time prediction of control strategies based on the monitoring of the water network. Though the control strategies for a number of time intervals within an optimization horizon are generated and transmitted back to field controllers at every time interval, only the ones for the current time interval are actuated in normal conditions, as the rest will be updated from the continuous optimization whenever a new set of monitoring data is made available from the field. The optimization methodology thus also possesses a high level of resilience in response to occasional data communication problems, in which case the following control strategies stored at the field controller other than the current ones can be actuated. Finally, simulation results based on the hydraulic model developed by Welsh Water have shown that the proposed methodology is able to reduce the network operational cost, with the proposed mixed-integer DE performing better than GAs. Furthermore, adaptive pricing was also suggested for all the pumping stations to allow more space for cost savings.

## Data accessibility

This article does not contain any additional data.
